# Association between sleep parameters and longitudinal shortening of telomere length

**DOI:** 10.18632/aging.203993

**Published:** 2022-04-02

**Authors:** Jeong-Hwa Jin, Hyuk Sung Kwon, Seong Hye Choi, Seong-Ho Koh, Eun-Hye Lee, Jee Hyang Jeong, Jae-Won Jang, Kyung Won Park, Eun-Joo Kim, Hee Jin Kim, Jin Yong Hong, Soo Jin Yoon, Bora Yoon, Hyun-Hee Park, Jungsoon Ha, Jong Eun Park, Myung Hoon Han

**Affiliations:** 1 Department of Neurology, Hanyang University College of Medicine, Guri, Republic of Korea; 2 Department of Neurology, Inha University School of Medicine, Incheon, Republic of Korea; 3 Department of Neurology, Ewha Womans University School of Medicine, Seoul, Republic of Korea; 4 Department of Neurology, Kangwon National University School of Medicine, Chuncheon, Republic of Korea; 5 Department of Neurology, Dong-A Medical Center, Dong-A University College of Medicine, Busan, Republic of Korea; 6 Department of Neurology, Pusan National University Hospital, Pusan National University School of Medicine and Medical Research Institute, Busan, Republic of Korea; 7 Department of Neurology, Samsung Medical Center, Sungkyunkwan University School of Medicine, Seoul, Republic of Korea; 8 Department of Neurology, Yonsei University Wonju College of Medicine, Wonju, Republic of Korea; 9 Department of Neurology, Eulji University Hospital, Eulji University School of Medicine, Daejeon, Republic of Korea; 10 Department of Neurology, Konyang University College of Medicine, Daejeon, Republic of Korea; 11 GemVax & Kael Co. Ltd., Seongnam, Republic of Korea; 12 Department of Laboratory Medicine, Hanyang University Guri Hospital, Hanyang University College of Medicine, Guri, Republic of Korea; 13 Department of Neurosurgery, Hanyang University Guri Hospital, Hanyang University College of Medicine, Guri, Republic of Korea

**Keywords:** sleep quality, telomere length, amyloid pathology, aging, sleep duration

## Abstract

Background: The relationship between sleep parameters and longitudinal shortening of telomere length is unclear. This study aimed to investigate the relationship between sleep parameters and the shortening of leukocyte telomere length (LTL) over a year.

Methods: Among the participants in the validation cohort of the Korea Brain Aging Study for the Early Diagnosis and Prediction of Alzheimer’s Disease, participants who measured both baseline and follow-up (two years later) of LTL were analyzed. They were dichotomized according to the degree of LTL attrition over two years. Clinical characteristics were compared between the faster and slower LTL shortening groups (cut-off points: −0.710 kbp, *n* = 119 each). Multivariable logistic regression analyses were performed to determine independent relationships between faster shortening of LTL length and sleep parameters.

Results: A total of 238 participants, aged 55–88 years, were included. Participants with faster LTL shortening had a shorter duration of sleep (*P* = 0.013) and longer sleep latency (*P* = 0.007). Among the components of the PSQI, subjective measures of sleep quality, sleep latency, sleep duration, and sleep efficiency were significantly worse in participants with faster LTL shortening. Multivariate logistic regression analysis showed that sleep duration (per hour, OR = 0.831, 95% CI = 0.698–0.989), sleep latency (per minute, OR = 1.013, 95% CI = 1.002–1.024), global PSQI score (OR = 1.134, 95% CI = 1.040–1.236), shortest sleep duration (OR = 5.173, 95% CI = 1.563–17.126), and lowest sleep efficiency (OR = 7.351, 95% CI = 1.943–27.946) were independently associated with faster LTL shortening.

Conclusions: Poor sleep quality, specifically short sleep duration, long sleep latency, and low sleep efficiency were associated with faster longitudinal shortening of LTL.

## INTRODUCTION

Telomeres are recurring sequences that are situated at the ends of eukaryotic chromosomes. They serve to protect the chromosome ends from being released in the process of cell division [1]. The length of the telomere shortens with age, but the attrition rate varies among individuals [2]. Leukocyte telomere length (LTL) is a widely used biomarker for physiological aging, which reflects aging-related diseases and environmental factors [3]. A shorter LTL is reported to be associated with chronological age, paternal age, male sex, ethnicity, psychiatric disorders (i.e., major depressive disorder and affective disorder), low physical activity, low socioeconomic status, heavy alcohol consumption, smoking, late sleep-onset, short sleep duration, and obstructive sleep apnea [4–9]. In terms of attrition rate, shortening of LTL is related to chronological age, male sex, baseline LTL, smoking, obesity, less exercise, perceived stress, and high childhood trauma index [4, 8]. However, the association between sleep parameters and attrition of LTL is unclear.

Sleep disturbances are common in the elderly, and prevalence increases as the population ages [10]. Shorter telomere length was associated with both sleep quality and duration. Sleep quality measured by the Pittsburgh Sleep Quality Index (PSQI) attenuated the relationship between age and telomere length [11]. For sleep duration, both shorter and longer sleep durations are related to increased all-cause mortality and cardiovascular events [12]. Similarly, short sleep duration was associated with shorter LTL [5, 11, 13], while long sleep duration (>9 hours) showed a marginal association with faster LTL attrition [8]. Although the pathophysiological mechanism between sleep parameters and LTL is unclear, sleep disturbance might be related to cellular aging and damage [14]. Inappropriate sleep duration can cause inflammation, oxidative stress, and neuroendocrine control disorders [15–18]. These factors might contribute to the relationship between sleep and LTL.

To our knowledge, no reports have shown a significant correlation between sleep parameters and accelerated attrition of LTL (faster shortening of telomere length over time). We aimed to investigate the relationship between sleep parameters (including sleep duration, sleep latency, sleep quality, sleep efficiency, sleep disturbance, sleep medications, and daytime sleep dysfunction) and LTL attrition over two years, using data from an independent validation cohort of the Korea Brain Aging Study for the Early Diagnosis and Prediction of Alzheimer’s Disease (KBASE-V). Initially, the correlation between LTL attrition and sleep parameters was analyzed. Then, participants were dichotomized according to the degree of LTL attrition, and we investigated factors that could independently predict the faster shortening of LTL.

## METHODS

### Participants

**Figure 1:**
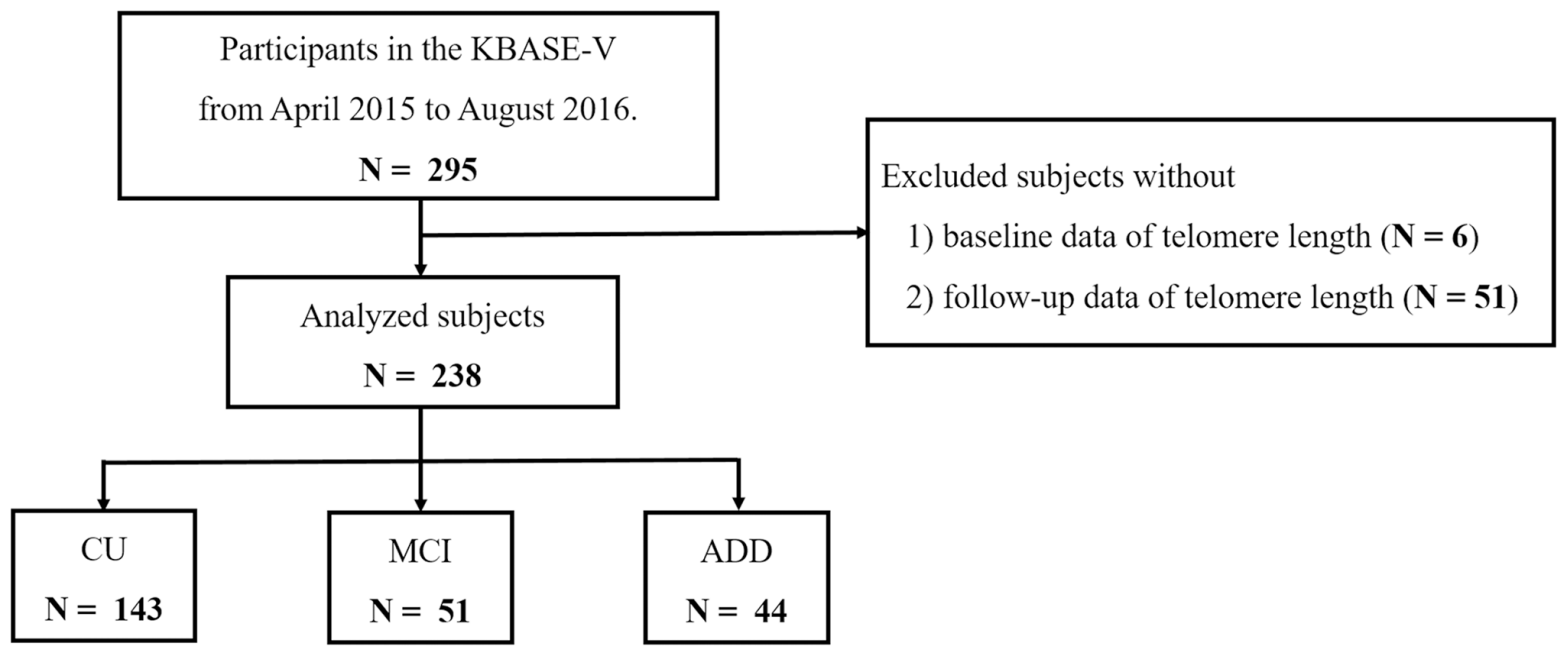
**Flowchart of the analyzed subjects.** Abbreviations: KBASE-V: Korea Brain Aging Study for the Early Diagnosis and Prediction of Alzheimer’s Disease; CU: cognitively unimpaired; MCI: mild cognitively impaired; ADD: Alzheimer’s disease dementia.

This study was performed by analyzing the data of participants in the KBASE-V, as described in a previous study [19], performed in accordance with the Good Clinical Practice guidelines and the Declaration of Helsinki, and approved by the institutional review board of each participating center (INHAUH 2015-03-021). The KBASE-V contains a nationwide cohort, including 167 cognitively unimpaired (CU), 72 mild cognitively impaired (MCI), and 56 Alzheimer’s disease dementia (ADD) participants from nine hospitals. All participants in the KBASE-V were aged 55–90 years. Each participant had a reliable informant, who provided investigators with required information. Each participant had a reliable informant who provided the investigators with required information. The exclusion criteria for this study included: (1) the presence of major psychiatric illness; (2) significant neurological or medical conditions, or comorbidities that could affect cognitive functions (except MCI or ADD); (3) contraindications for magnetic resonance imaging (MRI) (e.g., pacemaker, claustrophobia); (4) illiteracy; (5) severe visual or hearing difficulty, or serious communication or behavioral problems that could make a clinical examination or brain scan difficult; (6) use of an investigational drug; and (7) pregnancy or breastfeeding. Participants were eligible for inclusion in this study if they had both baseline and follow-up LTL measurements. We included 238 participants (143 CU, 51 MCI, and 44 ADD) in this study (Figure 1).

### Clinical assessment including sleep parameters

All participants underwent physical and neurological examinations, including thorough diagnostic procedures that assessed participants’ cognition, abnormal behaviors, activities of daily living (ADL), demographic characteristics, family history, current medications, vascular risk factors (the presence of hypertension, diabetes, dyslipidemia, and smoking status), and other comorbidities using the Mini-Mental State Examination (MMSE) [20], Geriatric Depression Scale (GDS) [21], Blessed Dementia Scale-ADL [22], clinical dementia rating scale [23], and Consortium to Establish a Registry for Alzheimer’s Disease yearly [19, 20]. Brain MRI and laboratory tests, such as APOE genotyping, were performed at baseline.

Hypertension was defined as systolic blood pressure higher than or equal to 140 mmHg, diastolic blood pressure higher than or equal to 90 mmHg, or use of antihypertensive medication [24]. Diabetes mellitus was defined based on current treatment with insulin or oral hypoglycemic medication, 8-hour fasting plasma glucose ≥126 mg/dL, or HbA1c levels ≥6.5% [25]. Dyslipidemia was defined as total cholesterol ≥200 mg/dL, low-density lipoprotein cholesterol ≥130 mg/dL, high-density lipoprotein cholesterol level <40 mg/dl, and triglyceride level ≥150 mg/dL, or the use of lipid-lowering drugs [26]. We measured the participants’ weight, height, and handgrip. Body mass index (BMI) was calculated as weight (kg) divided by the square of height (m^2^).

The Pittsburgh Sleep Quality Index (PSQI), Stanford Sleepiness Scale (SSS), and Epworth Sleepiness Scale (ESS) were used for sleep evaluation. Global PSQI score greater than five was considered as poor quality of sleep [27], and ESS score greater than or equal to eleven was considered as excessive day time sleepiness [28]. The SSS (scores ranging from 1 to 7) was used to quantify the progressive steps in daytime sleepiness [29]. Based on the PSQI, each participant was instructed to report their respective subjective sleep quality, sleep duration, sleep latency, habitual sleep efficiency, use of sleeping medication, sleep disturbances, and daytime dysfunction during the last month [27]. We used bioelectrical impedance analysis to measure the appendicular skeletal muscle mass index (ASMI) and proportion of fat.

### Brain MRI

All participants underwent brain MRI; a 3.0 T magnetic resonance scanner was used to capture 3D T1- and T2-weighted SPACE sagittal images with 0.8-mm thickness. Alzheimer’s disease neuroimaging initiative phase 2 MRI protocols were used in the brain MRI of this study [19, 30]. The 3D T1-weighted MRI parameters were as follows: repetition time (TR) = 2300 ms, echo time (TE) = 2.14 ms, inversion time (TI) = 900 ms, flip angle (FA) = 9°, and voxel resolution = 0.8 × 0.8 × 0.8 mm^3^, in the Skyra and Trio Tim scanners (Siemens, Washington, DC, USA); TR = 7.32 ms, TE = 3.02 ms, TI = 400 ms, FA = 11°, and voxel resolution = 0.8 × 0.8 × 0.8 mm^3^, in the General Electric (GE) Discovery MR750 scanner (GE Healthcare, Milwaukee, WI, USA); and TR = shortest (6.8 ms), TE = shortest (3.1 ms), FA = 9°, and voxel resolution = 0.8 × 0.8 × 0.8 mm^3^, in the Achieva scanner (Philips Healthcare, Andover, MA, USA).

The measured MRI data were analyzed as described previously using CIVET pipeline version 2.1, (http://mcin-cnim.ca/neuroimagingtechnologies/civet/) [19, 31]. The corrected T1-weighted images were segmented into the left and right sides of the hippocampus using FMRIB’s integrated registration and segmentation tool [32]. The volumes of the hippocampus were normalized to the total intracranial volume.

### Positive amyloid pathologic change (amyloid β biomarkers)

Amyloid pathologic change was considered positive when individuals displayed an abnormal Aβ biomarker considered by cortical amyloid positron electron tomography (PET) ligand binding and/or low cerebrospinal fluid (CSF) Aβ42 [33]. Of the total 199 participants in this study, 159 (79.9%) underwent amyloid PET at baseline. Sixty participants underwent ^11^C-PiB PET and ninety-nine underwent ^18^F-flutemetamol PET. CSF was collected from 100 participants (50.3%). In total, 184 (92.5%) participants were tested for Aβ biomarkers and 49 (24.6%) were positive.

The PET methods for each tracer and CSF analysis have been previously described [19, 34]. The standard uptake value ratio (SUVR) was obtained using the pons as a reference region on ^18^F-flutemetamol PET and the cerebellar gray matter as the reference region on ^11^C-PiB PET. Composite SUVR values were obtained by averaging the SUVR values for the frontal, temporal, parietal, occipital, anterior cingulate, and posterior cingulate/precuneus cortices. Based on previous work, elevated Aβ PET was defined as a composite SUVR higher than or equal to 0.634 on ^18^F-flutemetamol PET, and a composite SUVR higher than 1.21 on ^11^C-PiB PET [33]. The levels of Aβ42, t-tau, and p-tau in the CSF were measured using the multiplex xMAP Luminex platform with INNO-BIA AlzBio3 kits (Fujirebio Europe, Ghent, Belgium) as described in detail in a previous paper [19]. Based on the previous work, participants who underwent CSF studies were deemed to have Alzheimer’s disease pathology when the CSF Aβ42 was 433.68 pg/ml or lower.

### Telomere length assay

LTL was examined at the time when blood is collected (baseline and after 1 year) from participants. DNA was extracted from whole blood using G-DEXTM IIb RBC lysis buffer and G-DEXTM IIb cell lysis buffer (Intron, MA, USA). DNA hydration was performed using 300 μL of DNA hydration solution (QIAGEN, Hilden, Germany). Telomere length (TL) analysis was carried out using a nonradioactive TeloTAGGG TL Assay (Roche Boehringer-Mannheim, Grenzach-Wyhlen, Germany), as described by the manufacturer. Approximately 2–4 μg of DNA from each sample was digested with Hinf I/RsaI enzyme mix and isolated by gel electrophoresis. DNA fragments were transferred to a nylon membrane (Millipore, Bedford, MA, USA) by Southern transfer and hybridized to a digoxigenin (DIG)-labeled probe specific for telomeric repeats. The membrane was incubated with DIG-specific antibodies conjugated to alkaline phosphatase, and the probe was visualized by chemiluminescence using an image analyzer (ImageQuant LAS 4000, GE Healthcare, Little Chalfont, UK). The mean telomeric repeat-binding factor lengths were determined by comparison with the molecular weight standards.

LTL was measured in all participants who provided blood samples. All LTLs were analyzed by two researchers who were blinded to the patient information. The intraclass correlation coefficient (ICC) of the two raters was 0.905, indicating excellent reliability.

### Statistical analysis

Sample distribution was tested using the Kolmogorov-Smirnov test for factors, including sleep parameters and LTL. Data on LTL, education, ASMI, hand grip, sleep duration, sleep latency, PSQI, and ESS score are presented as median (interquartile range) as they did not present with normal distribution. Participants were grouped by initial diagnosis, sleep duration, sleep latency, and sleep efficacy, according to PSQI. Changes in LTL were compared among the groups using the Kruskal-Wallis test. When statistically significant overall differences were detected, the Mann-Whitney *U* test with Bonferroni correction was performed to analyze differences among subgroups. Within each group, differences in LTL between baseline and follow-up (two years later) were compared using the Wilcoxon signed-rank test (Supplementary Figures 1–4). Nonparametric correlations between sleep parameters and changes in LTL were calculated using Spearman’s test. All LTL levels were measured twice by independent researchers to reduce the expected error in measurements. The ICC was calculated to assess the reliability of LTL measurement. The ICC values between 0.75 and 0.9 indicate good reliability and values higher than 0.9 indicate excellent reliability [35]. The average LTL of the two raters was used in this study. As there are no criteria for faster shortening of LTL, the median value of LTL shortening (cut-off point: −0.710 kbp) was used to dichotomize the participants. Participants with faster shortening of LTL were considered as group 1, while those with slower shortening of LTL as group 2. We compared two groups using Pearson’s chi-square test for categorical variables and Student *t*-test and the Mann-Whitney *U* test for continuous variables. To assess the relationship between various parameters, including sleep factors and changes in LTL, logistic regression analyses were performed. Adjusted variables were age, sex, and factors selected from the results of the univariate analysis [*P* < 0.1 in univariate analysis (dyslipidemia and osteoporosis) (model 1)]. We additionally adjusted for the average of baseline and follow-up LTL in the final analysis (model 2). Statistical significance was set at a two-tailed *p-*value of < 0.05. All statistical analyses were performed using SPSS for Windows (version 21.0; SPSS Inc., Chicago, IL, USA).

## RESULTS

**Figure 2:**
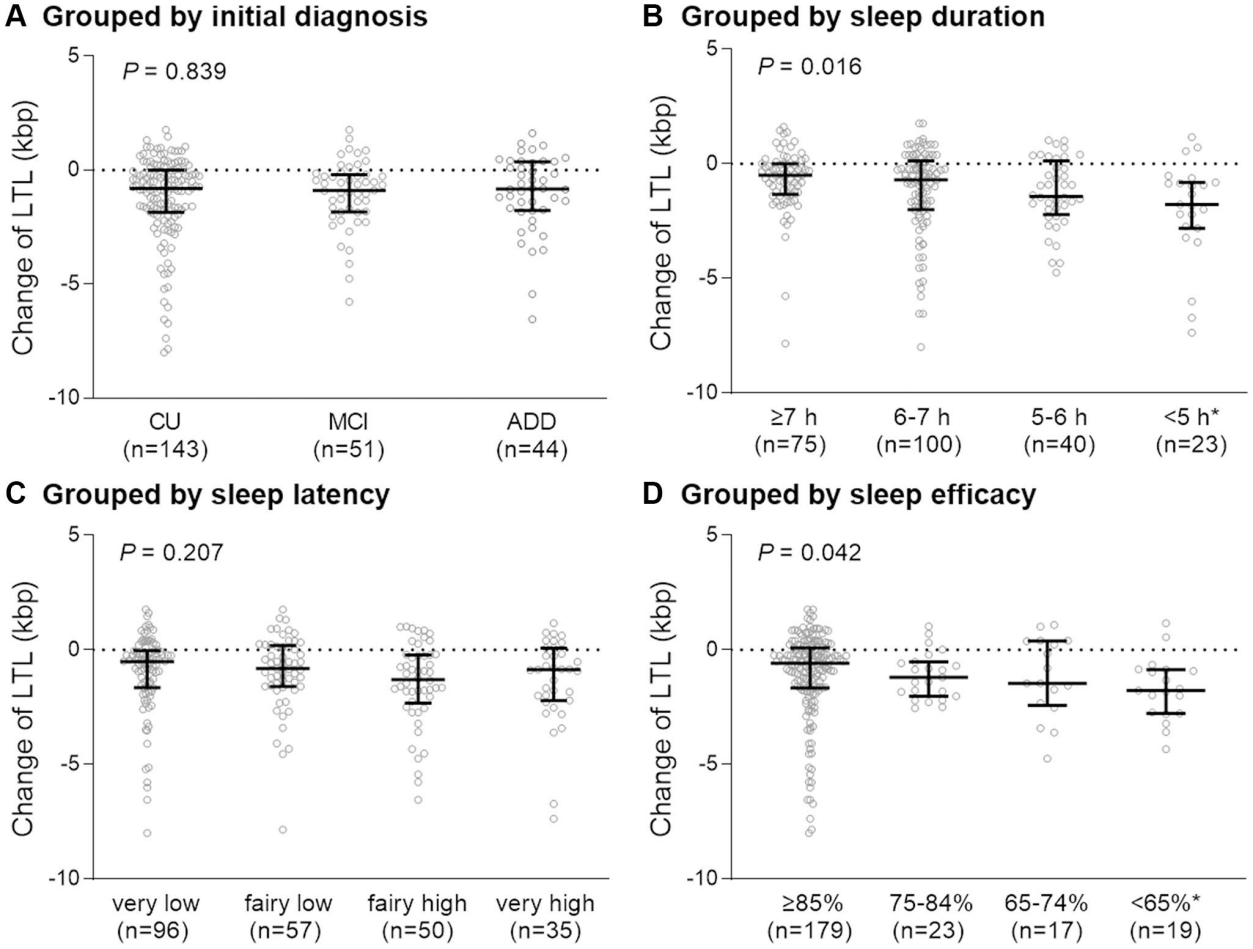
**Degree of change in telomere length over two years in each group.** Participants were grouped according to initial diagnosis (**A**), sleep duration (**B**), sleep latency (**C**), and sleep efficacy (**D**). Abbreviations: LTL: leukocyte telomere length; CU: cognitively unimpaired, MCI: mild cognitively impaired; ADD: Alzheimer’s disease dementia; h: hours. *P*-value for the Kruskal-Wallis test. ^*^P < 0.05 compared with the first group (sleep ≥7 h or sleep efficiency ≥85%).

A total of 238 (143 CU, 51 MCI, and 44 ADD) patients having both baseline and follow-up LTL measurements and were included in this study. Among them, 143 (60.1%) were women. Participants’ ages ranged from 55 to 88 years, with a mean ± SD of 70.2 ± 8.2 years. Overall, the average BMI was 24.2 ± 3.1 kg/m^2^ and the global PSQI score was 7.6 ± 3.6. The mean duration of sleep was 6.4 ± 1.7 hours and sleep latency (mean ± SD) was 26.7 ± 28.9 minutes. Baseline LTL (mean ± SD) was 7.82 ± 1.81 (CU: 8.01 ± 2.00, MCI: 7.57 ± 1.32, AD: 7.53 ± 1.55) kbp and follow-up LTL was 6.69 ± 0.85 (CU: 6.808 ± 0.85, MCI: 6.53 ± 0.84, AD: 6.53 ± 0.81) kbp. Shortening of LTL was significantly higher in patients who sleep <5 hours than in those who sleep ≥7 hours (Figure 2B). Also shortening of LTL was higher in patients with sleep efficiency <65% than in those with sleep efficiency ≥85% (Figure 2D). However, there were no significant differences in LTL shortening based on initial diagnosis or sleep latency (Figure 2A, 2C).

Participants were dichotomized according to the shortening of LTL and compared as follows: faster shortening of LTL (group 1) vs. slower shortening of LTL (group 2). The baseline demographic and clinical characteristics of the two groups are shown in Table 1. Osteoporosis was more prevalent in group 1. Participants in group 1 had longer baseline LTL and shorter follow-up LTL. Other factors, including cognitive stage, being a current smoker, past medical history (except osteoporosis), positive amyloid pathology, APOE e4 carrier status, MMSE score, GDS score, exercise status, and power of handgrip, did not differ between the two groups.

**Table 1:** Baseline characteristics of patients according to attrition of LTL.

	**Attrition of LTL**		***p***
	**Faster, group 1 (*n* = 119)**	**Slower, group 2 (*n* = 119)**	
Demographics			
Age, years	70.5 ± 8.0	69.9 ± 8.6	0.583
Sex, female (%)	77 (64.7)	66 (55.5)	0.145
Live alone	18 (15.3)	17 (14.5)	0.876
BMI, kg/m^2^	24.2 ± 2.8	24.2 ± 3.1	0.974
Education, years (IQR)	9.0 (6.0, 13.0)	10.0 (6.0, 13.0)	0.783^*^
Baseline telomere length (kbp)	8.215 (7.355, 9.815)	6.87 (6.375, 7.16)	<0.001^*^
Follow up telomere length (kbp)	6.265 (5.79, 6.95)	6.84 (6.365, 7.465)	<0.001^*^
Change of telomere length (kbp)	−1.81 (−2.805, −1.25)	−0.005 (−0.415, 0.475)	<0.001^*^
Cognitive stage			0.987
Cognitive unimpaired (%)	71 (59.7)	72 (60.5)	
Mild cognitive impairment (%)	26 (21.8)	25 (21.0)	
Dementia (%)	22 (18.5)	22 (18.5)	
Medical History			
Hypertension^**^	53/118 (44.9)	53/118 (44.9)	1.000
Diabetes mellitus	17 (14.3)	20 (16.8)	0.591
Dyslipidemia^**^	47/115 (40.9)	36/118 (30.5)	0.099
Coronary artery disease^**^	7/119 (5.9)	6 (5.1)	0.787
Cerebrovascular disease	4 (3.4)	7 (5.9)	0.354
Osteoporosis	20 (16.8)	9 (7.6)	0.029
Osteoarthritis	25 (21.0)	19 (16.0)	0.378
Current smoker	3 (2.5)	4 (2.5)	0.667
Taking more than three pills	67 (56.8)	70 (59.3)	0.692
Hippocampal volume, cm^3^	48.0 ± 9.2	48.6 ± 8.6	0.598
Positive amyloid pathology	41 (41.4)	35 (35.7)	0.411
APOE ε4 carrier	36 (30.3)	25 (21.0)	0.102
MMSE score, median (IQR)	26.0 (22.0, 29.0)	25·0 (21.0, 28.0)	0.150^*^
CDR score, median (IQR)	0.0 (0.0, 0.5)	0·0 (0.0, 0.5)	0.887^*^
CDR-SOB score, median (IQR)	0.0 (0.0, 1.0)	0·0 (0.0, 1.0)	0.829^*^
GDS score, median (IQR)	8.0 (4.0, 14.0)	8·0 (4.0, 12.5)	0.708^*^
Active exercise^†^	20 (16.8)	23 (19.3)	0.613
Passive exercise^†^	27 (22.7)	30 (25.2)	0.649
Meeting the PA guideline^‡^	28 (23.5)	28 (23.5)	1.000
ASMI, kg/m^2^ (IQR)	8.42 (7.85, 9.68)	8.70 (7.87, 9.89)	0.283^*^
Percentage of body fat, %	30.6 ± 7.2	30.4 ± 7.2	0.859
Handgrip, kg (IQR)	24.9 (19.6, 30.8)	24.5 (19.3, 32.3)	0.942^*^
MNA (IQR, range: 0–30)	25.0 (23.0, 26.0)	24.5 (23.0, 26.0)	0.752^*^

Data are presented as mean ± SD or number (%), unless otherwise indicated. Abbreviations: LTL: leukocyte telomere length; MMSE: Mini-Mental State Examination; IQR: interquartile range; CDR-SOB: Clinical Rating Scale Sum of Boxes; GDS: Geriatric Depression Scale; PA: physical activity; ASMI: appendicular skeletal muscle mass index; MNA: Mini Nutritional Assessment. ^*^Missing values are presented. Student’s *t-*test, Pearson’s chi-square test, and ^**^Mann-Whitney *U* test were used. ^†^Once or more during the last one week. ^‡^≥600 metabolic equivalent minutes of physical activity per week.

The sleep parameters in relation to the degree of longitudinal shortening of LTL are shown in Table 2. Group 1 had a shorter duration of sleep (*P* = 0.013) and longer sleep latency (*P* = 0.007). Among the components of the PSQI, subjective measures of sleep quality (*P* = 0.037), sleep latency (*P* = 0.014), sleep duration (*P* < 0.001), and sleep efficiency (*P* < 0.001) were significantly worse in group 1. Other factors, including sleep disturbance, sleep medication, daytime sleep dysfunction, ESS score, and SSS score, were not different between the two groups.

**Table 2:** Sleep parameters in relation to longitudinal shortening of LTL.

	**Attrition of LTL**		***p***
	**Faster (*n* = 119)**	**Slower (*n* = 119)**	
Duration of sleep, hour (IQR)	6.0 (5.0, 7.0)	7.0 (6.0, 8.0)	0.008^*^
Sleep latency, minute (IQR)	25.0 (10.0, 30.0)	10.0 (5.0, 30.0)	0.016^*^
PSQI			
Global score (range: 0–21)	7.0 (5.0, 11.0)	6.0 (5.0, 8.0)	0.002^*^
Sleep quality (IQR, range: 0–3)	2.0 (2.0, 3.0)	2.0 (2.0, 2.0)	0.037^*^
Sleep latency (IQR, range: 0–3)	1.0 (0.0, 2.0)	1.0 (0.0, 2.0)	0.014^*^
Sleep duration (IQR, range: 0–3)	1.0 (1.0, 2.0)	1.0 (0.0, 1.0)	<0.001^*^
Sleep efficiency (IQR, range: 0–3)	0.0 (0.0, 1.0)	0.0 (0.0, 0.0)	0.001^*^
Sleep disturbance (IQR, range: 0–3)	1.0 (1.0, 1.0)	1.0 (1.0, 1.0)	0.269^*^
Sleep medication (IQR, range: 0–3)	0.0 (0.0, 0.0)	0.0 (0.0, 0.0)	0.749^*^
Daytime sleep dysfunction (IQR, range: 0–3)	1.0 (1.0, 2.0)	1.0 (1.0, 2.0)	0.307^*^
Poor quality sleep (PSQI > 5)	86 (72.3)	73 (61.3)	0.074^†^
ESS score (IQR)	4.0 (2.0, 5.0)	4.0 (2.0, 6.0)	0.350
Excessive daytime sleepiness (ESS > 11)	6 (5.0)	5 (4.2)	0.758^†^
Stanford Sleepiness Scale (IQR)	2.0 (1.0, 3.0)	2.0 (1.0, 2.0)	0.734^*^
Severe snoring, ≥1/week	73 (61.3)	59 (49.6)	0.068^†^
Witnessed apnea, ≥1/week	72 (60.5)	59 (49.6)	0.090^†^

Data are presented as mean ± SD, number (%), or indicated. Abbreviations: LTL: leukocyte telomere length; IQR: interquartile range; PSQI: Pittsburgh Sleep Quality Index; ESS: Epworth sleepiness scale. Student’s *t-*test, ^†^Pearson chi-square test, and ^*^Mann-Whitney *U* test were use.

Multivariate logistic regression analysis after adjusting for age, sex, osteoporosis, and dyslipidemia showed that sleep duration (odds ratio [OR] = 0.829, 95% confidence interval [CI] = 0.701–0.982, 90% CI = 0.720–0.955, *P* = 0.030), sleep latency (OR = 1.012, 95% CI = 1.001–1.022, 90% CI = 1.003–1.021, *P* = 0.030), and global PSQI score (OR =1.117, 95% CI = 1.030–1.213, 90% CI = 1.043–1.197, *P* = 0.008) predicted faster LTL shortening. Among the PSQI, fairly bad to very good subjective sleep quality ratio (OR = 2.623, 95% CI = 1.054–6.527, 90% CI = 1.221–5.637, *P* = 0.038), shortest to longest sleep duration ratio (OR = 5.587, 95% CI = 1.781–17.529, 90% CI = 2.140–14.586, *P* = 0.003), 5–6 hours to longest sleep duration ratio (OR = 2.774, 95% CI = 1.201–6.408, 90% CI = 1.374–5.601, *P* = 0.017), and lowest to highest sleep efficiency ratio (OR = 6.093, 95% CI = 1.662–22.339, 90% CI 2.048–18.128, *P* = 0.006) were associated with faster LTL shortening (Table 3).

**Table 3:** Multivariable logistic regression analysis predicting faster shortening of telomere length by sleep parameters.

	**Unadjusted OR**	**Model 1**	***p***	**Model 2**	***p***
Sleep duration, per 1 hour	0.818 (0.696–0.962)	0.829 (0.701–0.982)	0.030	0.831 (0.698–0.989)	0.037
Sleep latency, per 1 minute	1.014 (1.003–1.025)	1.012 (1.001–1.022)	0.030	1.013 (1.002–1.024)	0.020
Global PSQI score	1.140 (1.055–1.233)	1.117 (1.030–1.213)	0.008	1.134 (1.040–1.236)	0.004
Sleep quality					
0 (very good)	1 [ref]	1 [ref]		1 [ref]	
1 (fairly good)	1.171 (0.566–2.420)	1.084 (0.516–2.279)	0.832	1.187 (0.533–2.644)	0.673
2 (fairly bad)	3.117 (1.286–7.554)	2.623 (1.054–6.527)	0.038	2.876 (1.080–7.661)	0.035
3 (very bad)	0.786 (0.196–3.145)	0.574 (0.134–2.465)	0.455	0.645 (0.135–3.096)	0.584
Sleep latency					
0 (very low)	1 [ref]	1 [ref]		1 [ref]	
1 (fairly low)	1.514 (0.782–2.929)	1.510 (0.764–2.987)	0.236	2.055 (0.964–4.385)	0.062
2 (fairly high)	2.385 (1.182–4.809)	1.950 (0.937–4.060)	0·074	1.982 (0.905–4.341)	0.087
3 (very high)	1.949 (0.890–4.266)	1.625 (0.716–3.688)	0.264	1.823 (0.775–4.290)	0.169
Sleep duration					
0 (≥7 h)	1 [ref]	1 [ref]		1 [ref]	
1 (6–7 h)	1.407 (0.765–2.585)	1.472 (0.774–2.801)	0.239	1.124 (0.559–2.259)	0.744
2 (5–6 h)	2.644 (1.199–5.831)	2.774 (1.201–6.408)	0.017	2.659 (1.098–6.438)	0.030
3 (<5 h)	5.710 (1.912–17.059)	5.587 (1.781–17.529)	0.003	5.173 (1.563–17.126)	0.007
Sleep efficiency					
0 (≥85%)	1 [ref]	1 [ref]		1 [ref]	
1 (75–84%)	2.373 (0.958–5.881)	2.124 (0.834–5.413)	0.114	3.072 (1.147–8.231)	0.026
2 (65–74%)	1.424 (0.525–3.859)	1.298 (0.467–3.605)	0.617	0.987 (0.322–3.021)	0.987
3 (<65%)	6.751 (1.900–23.990)	6.093 (1.662–22.339)	0.006	7.351 (1.934–27.946)	0.003
Osteoporosis	2.469 (1.074–5.675)	2.288 (0.961–5.445)	0.061	2.197 (0.884–5.462)	0.090

*p* for multivariate models. Data are presented as odds ratios (95% confidence interval). Model 1: Adjusted for age, sex, osteoporosis, and dyslipidemia. Model 2: Additionally adjusted for the average of baseline and follow-up leukocyte telomere length (LTL).

After additional adjustment for the average of baseline and follow-up LTL, sleep duration (OR = 0.831, 95% CI = 0.698–0.989, 90% CI = 0.718–0.961, *P* = 0.037), sleep latency (OR = 1.013, 95% CI = 1.002–1.024, 90% CI = 1.004–1.022, *P* = 0.020), global PSQI score (OR = 1.134, 95% CI = 1.040–1.236, 90% CI = 1.054–1.219, *P* = 0.004), shortest to longest sleep duration ratio (OR = 5.173, 95% CI = 1.563–17.126, 90% CI = 1.894–14.128, *P* = 0.007), 5–6 hours to longest sleep duration ratio (OR = 2.659, 95% CI = 1.098–6.438, 90% CI = 1.266–5.585, *P* = 0.030), and lowest to highest sleep efficiency ratio (OR = 7.351, 95% CI = 1.934–27.946, 90% CI = 2.397–22.547, *P* = 0.003) were independently associated with faster LTL shortening.

## DISCUSSION

This study demonstrated that a higher global PSQI score, shorter sleep duration, and longer sleep latency were consistently associated with faster LTL shortening. Among the components of the PSQI, the shortest sleep duration (<5 hours), and lowest sleep efficiency (<65%) were significantly associated with faster shortening of LTL. To our knowledge, this study is the first to show a significant relationship between sleep parameters and accelerated attrition of LTL.

Previous cross-sectional studies that examined the association between sleep parameters and LTL showed conflicting results. From the Whitehall II cohort study, Jackowska and colleagues found a significant linear relationship between sleep duration and LTL in men but not in women [13]. Sleep duration of at least 7 hours had longer LTL than those with sleep duration of less than 7 hours, in patients with human immunodeficiency virus [36]. However, sleep quality was not associated with LTL [36]. Sleep duration or sleep quality did not predict LTL on their own in a sample of middle-aged to older adults, but they moderated the relationship between age and LTL [11]. However, another study showed that poor sleep quality predicts shorter LTL in women aged 49–66 years [37]. Sleep quality was related to shorter TL of specific immune cells in obese individuals [38]. In children, shorter sleep duration was associated with shorter LTL [17]. Insomnia was associated with short LTL at age 70–88 years, but this was not significant at age 60–69 years [14]. The reason the results differ between studies seems to be that diverse factors influence the attrition rate of telomere length, and the degree of this influence may differ according to age, sex, and comorbidities of participants.

A recent report analyzed the association between sleep parameters and attrition of LTL over six years using data from the Netherlands Study of Depression and Anxiety (NESDA) [5]. However, they could not find any sleep parameters that predicted faster shortening of LTL [5]. Including older individuals (initial mean age ± SD of the current study vs. the NESDA, 70.2 ± 8.2, vs. 41.8 ± 13.1 years) in our study might reveal a significant relationship between sleep parameters and changes in LTL over time.

The biological mechanisms linking sleep quality and LTL need to be further elucidated. However, inflammation, oxidative stress, increment of sympathetic tone, and secretion of cortisol are possible mechanisms that explain the association between them. Sleep deprivation and disrupted sleep quality increase inflammatory cytokines and oxidative stress [39, 40]. Accumulation of inflammatory burden and oxidative stress may increase LTL loss [41]. Oxidative stress is also known to be associated with premature aging and shortening of LTL [42]. Sleep disturbance has effects on autonomic dysfunction [16], catecholamine level [43], and cortisol secretion [44], which might also contribute to the shortening of LTL [45].

There are some limitations in our study. First, objective measurements, such as actinography, polysomnography, melatonin level, or telomerase activity, were not analyzed. Therefore, we could not evaluate obstructive sleep apnea, which is known to be associated with LTL [7]. Although PSQI is well validated and self-reported sleep duration is similar to objective measurements [38, 46], future studies should consider including objective measurements. Second, the two-year follow-up period may be too short to reveal a significant relationship between LTL attrition and other factors (such as osteoporosis and APOE ε4 carrier status), and the number of patients was small. There is a possibility that the expected error in the measurement is higher than the expected shortening of telomere length over two years. To reduce measurement errors, independent researchers analyzed all telomere lengths twice. In addition, to minimize the effect of measurement error and overcome the fact that LTL did not follow a normal distribution, we dichotomized the participants according to the shortening of LTL. It is encouraging that sleep duration, latency, and efficacy showed statistically significant associations with changes in LTL over just two years. Third, participants were of a single ethnicity (Korean) with old age and a high proportion (39.9%) of cognitive impairments. Therefore, caution should be taken when extrapolating the results of the current study to the general population.

In conclusion, poor sleep quality measured by the PSQI score, specifically, long sleep latency, short sleep duration (<5 hours), and low sleep efficiency (<65%), are associated with accelerated shortening of LTL. These results suggest that sleep latency, sleep duration, and sleep efficiency may be added as the modifiable risk factors associated with cellular aging. Further longitudinal studies with longer follow-up durations and measurements of objective markers may clarify these results. If these associations are further demonstrated, such interventions (i.e., sleep hygiene interventions and medical treatments) to improve sleep quality might attenuate the shortening of telomere length.

## Supplementary Materials

Supplementary Figures
