# Supplementary material for: Association between sleep parameters and longitudinal shortening of telomere length
Source: Aging (Albany NY). 2022 Apr 2;14(7):2930–44. doi: 10.18632/aging.203993 (PMC9037260; doi:10.18632/aging.203993)
Supplement: Supplementary Figures [file aging-14-203993-s001.pdf]

SUPPLEMENTARY FIGURES

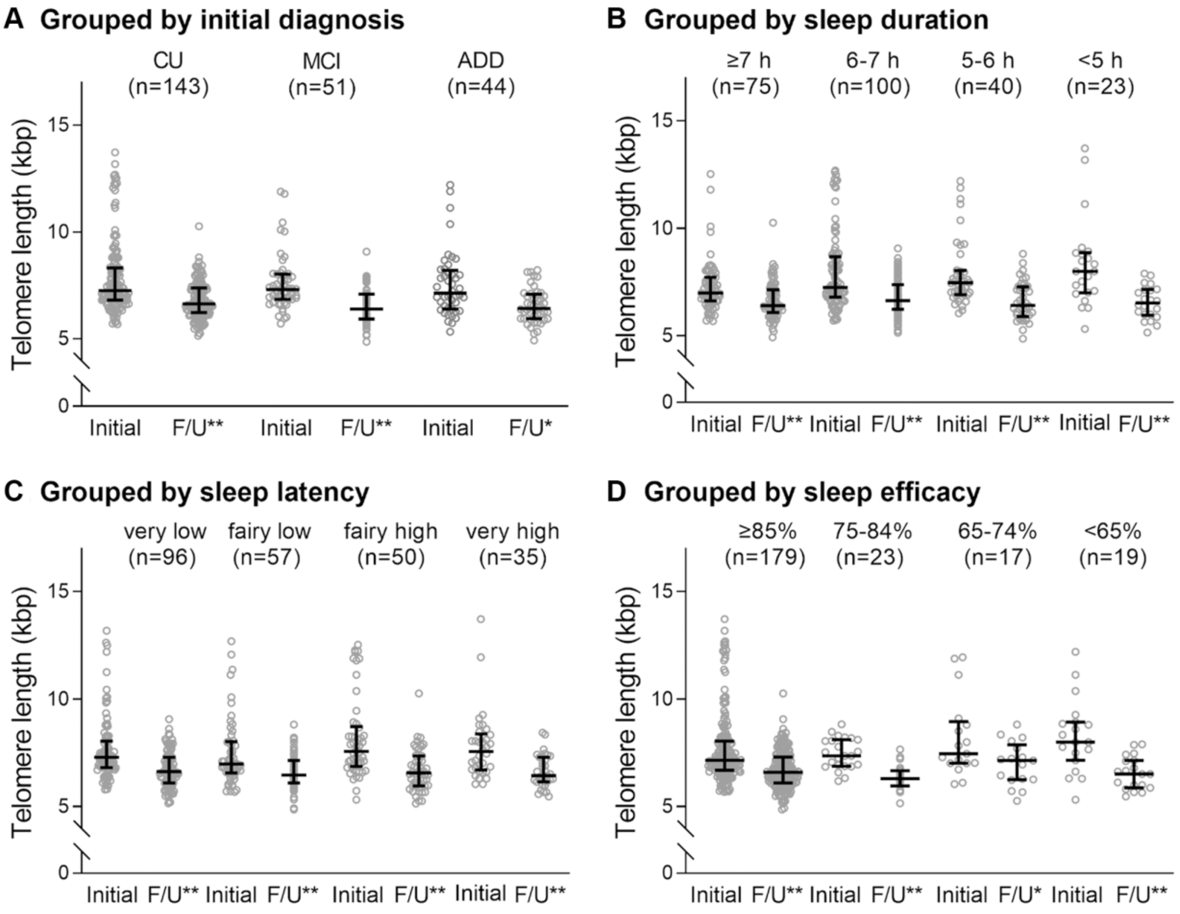

Supplementary Figure 1. Initial and follow up leukocyte telomere length in the current study. Participants were grouped according to initial diagnosis (**A**), sleep duration (**B**), sleep latency (**C**), and sleep efficacy (**D**). Abbreviations: CU: cognitively unimpaired; MCI: mild cognitive impairment; ADD: Alzheimer's disease dementia; h: hours. *P*-value for the Kruskal-Wallis test. $^{*}P$ < 0.05 and $^{**}P$ < 0.01 compared to initial telomere length.

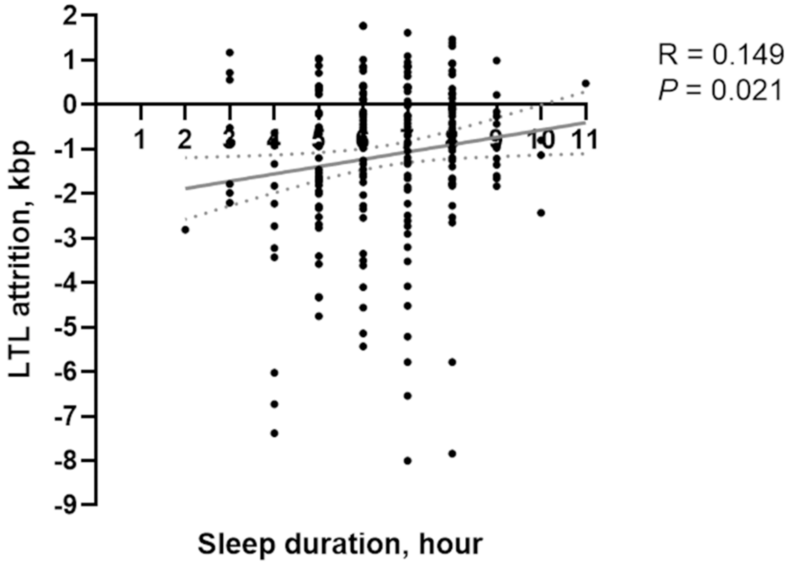

Supplementary Figure 2. The correlation between baseline sleep duration and attrition of leukocyte telomere length. Correlation coefficient (R) and *P*-value were analyzed using Spearman's bivariate correlation.

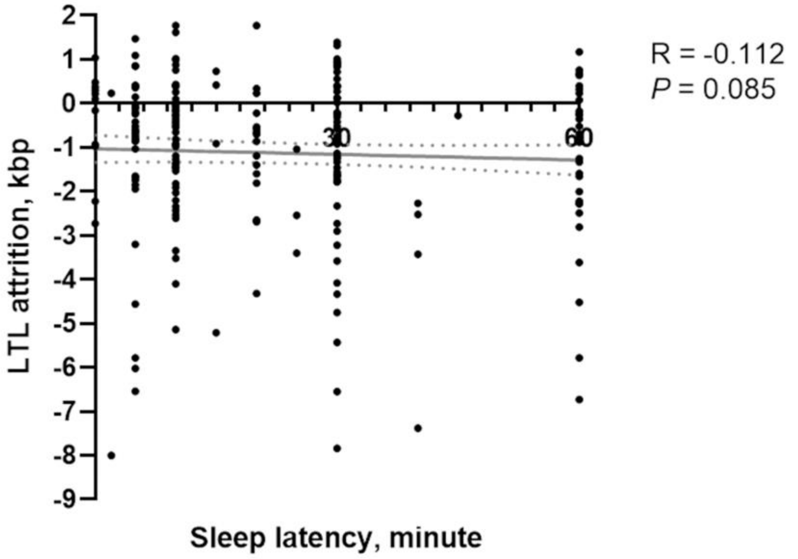

Supplementary Figure 3. Correlation between baseline sleep latency and attrition of leukocyte telomere length. The correlation coefficient (R) and *P*-value were analyzed using Spearman's bivariate correlation.

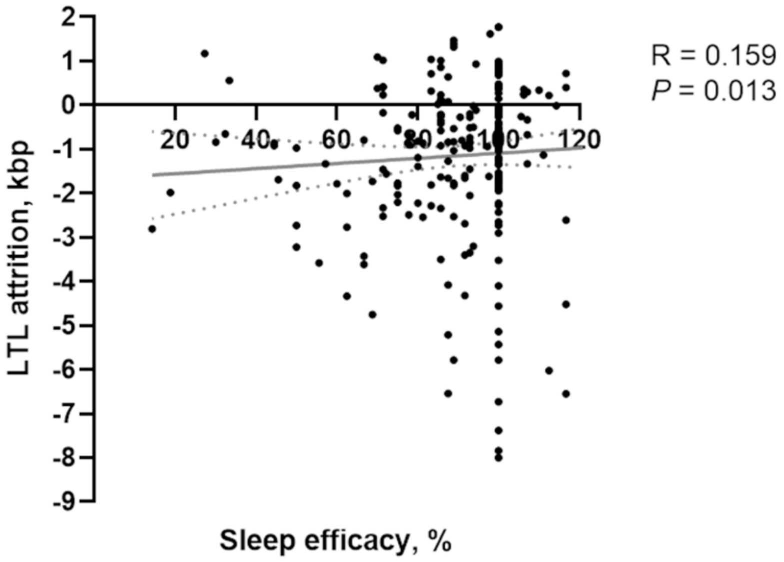

Supplementary Figure 4. Correlation between baseline sleep efficacy and attrition of leukocyte telomere length. The correlation coefficient (R) and *P*-value were analyzed using Spearman's bivariate correlation.
